# Microbiological Efficacy and Cost-Effectiveness of Poultry Carcass Excision Sampling Methods in Brazil

**DOI:** 10.3390/microorganisms14020372

**Published:** 2026-02-05

**Authors:** Pricila Borges, Luciana Mena, Sandra Heidtmann, José Queluz, Natalia Lopes, Jaqueline Cruvinel, Michele Nesi, Juliana Schmitz, Anabile Lisboa, Viviane Colla, Christiane Huller, Brunna Dutra, Eduardo Tondo

**Affiliations:** 1 Food Company, São Paulo 04730-090, SP, Brazilluciana.mena@unesp.br (L.M.); anabilecrotti6@gmail.com (A.L.);; 2 Instituto de Ciência e Tecnologia de Alimentos, Universidade Federal do Rio Grande do Sul—ICTA/UFRGS, Porto Alegre 91501-970, RS, Brazil

**Keywords:** chicken meat, *Enterobacteriaceae*, neck skin

## Abstract

Brazil is among the world’s leading exporters of chicken meat, and microbiological evaluation of carcasses is essential to verify process hygiene and safety. This study assessed the microbiological effectiveness and economic impact of two sampling methods for poultry carcasses: the excision of pooled samples of skin and muscle from multiple carcass regions, as recommended by Brazilian authorities, and the excision of neck skin alone. In accordance with Brazilian authorities guidelines requiring carcass evaluation through *Enterobacteriaceae* counts, these microorganisms were employed to assess contamination across different regions of 90 carcasses. Subsequently, *Enterobacteriaceae* counts were performed on 144 carcasses using both sampling methods. Mesophilic microorganisms, total coliforms, *Escherichia coli*, and *Staphylococcus* were tested in ten carcasses sampled by both methods to confirm the results obtained from the enumeration of *Enterobacteriaceae* and results were evaluated using Shapiro–Wilk, Levene, F-test, Kruskal–Wallis, Dunn, and *T*-test. Additionally, costs related to labor time and protein waste were quantified in 18 slaughterhouses. Results showed that *Enterobacteriaceae* counts in neck, cloaca, and wing regions were similar but significantly lower than those in pooled dorsal samples (*p* < 0.05). Neck skin samples were statistically comparable to dorsal pooled samples and exhibited higher contamination than ventral samples, demonstrating equivalent or superior microbiological representativeness. The neck skin method required less time, produced less protein waste, and reduced costs by 99%, indicating a more efficient and cost-effective alternative for microbiological monitoring of poultry carcasses.

## 1. Introduction

Chicken meat ranks among the most widely consumed animal proteins globally, with Brazil standing as the third-largest producer and the leading exporter of this commodity [1]. Within this huge framework, thousands of chicken carcasses are microbiologically tested every day, necessitating the use of methods that are both effective and economically feasible.

Microbiological evaluation of chicken carcasses has been used to assess the prevalence of pathogens [2,3], contamination by indicator microorganisms at specific stages of a process [4,5], or to monitor the performance of production processes as a whole [6]. Sampling methods for these carcasses may have different performances [7,8], affecting decision-making and trading.

According to ISO 17604:2015 [9], which specifies methods for sampling carcasses for microbiological analysis, the choice of sampling method depends mainly on the purpose of the analysis, the required sensitivity, and the practical aspects of sampling, such as collection methods. According to this standard, corroborated by Zhang et al. [10], skin excision, swabbing, and whole carcass washing are three commonly used methods for sampling poultry carcasses. In general, excision methods collect portions of skin and muscle or just skin from a specific part or different parts of the carcasses, while the swab method uses cotton swabs or sponges to swab the surfaces of the carcasses. In the rinsing method, an entire chicken carcass is placed inside a plastic bag, and then enough diluent is added to rinse it thoroughly. An aliquot of the rinse is seeded onto plates containing culture media for microbial counting after incubation.

Studies report that similar amounts of microorganisms were collected by the excision and rinsing methods [7,8], but the latter method requires a larger amount of diluents and large plastic bags, which can generate very high costs when analyzing a large number of samples. These and other studies [7,8,9] agree that swab sampling methods tend to collect fewer microorganisms and are more difficult to standardize than other methods, which are considered important disadvantages of this method. Sharpe et al. [11] reported that excision methods can collect a greater number of microorganisms from the surface of carcasses when compared to rinsing and swab methods, suggesting that excision sampling would be more appropriate for the microbiological analysis of chicken carcasses.

Excision methods can be performed in different ways, depending on the sampling technique used. Methods that collect samples from various parts of the carcasses are difficult to perform on the production line, often requiring the carcasses to be collected and transported to laboratories where they will be sampled and analyzed. In addition to the costs and labor involved in transporting these carcasses, the samples need a lot of space in laboratories, and collecting excisions generates a lot of work for laboratory technicians. Furthermore, this sampling method is destructive, generating a large amount of poultry meat waste if sampling is frequent.

An alternative to this method would be to collect skin excisions only from the most contaminated region of the carcass, which must be identified before sampling. Ideally, collection from this single region should be carried out inside the slaughterhouse, avoiding the waste of whole carcasses and higher costs for transportation and collection material.

In Brazil, on 29 February 2024, Ordinance 1023 of the Ministry of Agriculture and Livestock (MAPA) came into force, approving procedures for the microbiological evaluation of the hygienic-sanitary performance of the broiler slaughtering process. Compliance with this regulation involved collecting chicken carcasses at the exit of each pre-cooling system (chillers) every hour of operation. The collected carcasses are packaged and sent refrigerated to laboratories where 25 g of skin and muscle from the pericloacal, neck, and wing regions of each carcass are collected and subjected to *Enterobacteriaceae* enumeration. These procedures were carried out throughout Brazil, generating a large volume of animal protein discarded daily, high costs related to transportation, labor, and analysis. Since this method has some disadvantages and there are other excision methods that could be as effective and representative as this one, the objective of the present study was to identify a possible alternative excision method and compare it with the excision method established by Ordinance 1023/2024 for microbiological analyses of chicken carcasses. In addition, the economic aspects involved in performing each method were also evaluated using quantifiable indicators.

## 2. Materials and Methods

### 2.1. Experimental Design and Location

The experiments were conducted from September 2024 to June 2025 in laboratories belonging to a large Brazilian chicken processing company and were divided into two stages. Stage 1 was carried out to verify whether specific carcass parts (cloaca, neck, and wings) would present different levels of microbiological contamination and whether pooled samples, collected from those different parts from the ventral and dorsal regions, would present differing contamination levels. *Enterobacteriaceae* counts were performed on these samples. The most contaminated and easiest-to-collect region of the carcasses was identified and chosen for the second stage of the study.

In Stage 2, skin and muscle samples from the cloaca, neck, and wing regions were collected and pooled, as established by Ordinance 1023/2024 [6], and then subjected to *Enterobacteriaceae* counts. These counts were compared to counts from the region identified in Stage 1 to verify their microbiological equivalence. The number of carcasses sampled by both excision methods was collected over five days in two large slaughterhouses in Brazil, following the criteria established by Ordinance 1023/2024. The cost of both sampling procedures was also compared. Details of each stage of the study are described below.

### 2.2. Microbiological Contamination Assessment of Carcass Parts (Stage 1)

For the Phase 1 tests, 90 chicken carcasses were collected at the exit of the chillers of each of the three slaughterhouses, allocated as follows: (i) 30 carcasses were sampled individually for the wing, neck, and cloaca regions; (ii) 30 carcasses were used to obtain ventral pooled samples (25 g of skin and muscle from the three ventral regions); and (iii) 30 carcasses were used to obtain dorsal pooled samples (25 g of skin and muscle from the three dorsal regions). This sampling enabled the comparison of the microbiological contamination of ventral and dorsal pooled samples. Poultry slaughterhouse A is located in the state of Santa Catarina and has two automatic chicken slaughter lines and two semi-automatic turkey slaughter lines, operating in two shifts with a slaughter capacity of 216,000 birds per day, which have an average clean weight of 2.11 kg. Slaughterhouse B is located in the state of Santa Catarina and has three slaughter lines operating in two shifts, with a slaughter capacity of 400,000 birds per day, which have an average clean weight of 2.11 kg. Slaughterhouse C is located in the midwestern part of the state of Santa Catarina, has two slaughter lines, operates in three shifts, with a slaughter capacity of 320,000 birds per day, which have an average clean weight of 1.04 kg. All samples were collected by a quality assurance employee from each slaughterhouse, wearing full uniforms and nitrile gloves. The collected carcasses were placed in sterilized plastic bags, packed in thermal boxes at 2 to 8 °C, and then quickly transported to the respective slaughterhouse laboratories.

In the laboratories, 25 g samples of skin and muscle from the wing region, 30 samples of skin and muscle from the neck region, and 30 samples of skin and muscle from the cloaca region were collected separately from 30 carcasses. From other 30 carcasses, 30 samples containing 25 g of skin and muscle were collected from the cloaca, neck, and wing regions of the ventral side of the carcasses, The samples collected from each carcass were put mixed. From other 30 carcasses, 30 samples containing 25 g of skin and muscle were collected from the cloaca, neck, and wing regions of the dorsal side of the carcasses. All samples were weighed on a calibrated scale inside sterile bags and subjected to *Enterobacteriaceae* enumeration, as described below.

### 2.3. Comparison of Microbiological Contamination of Samples Collected by Two Excision Methods (Stage 2)

One hundred and forty-four (144) chicken carcasses were collected at the exit of the chillers of two large Brazilian slaughterhouses. This number of samples was representative of the number of samples that should be collected from these two slaughterhouses during five working days, in accordance with Ordinance 1023/2024. The slaughterhouses have the following characteristics: Poultry slaughterhouse D is located in the state of Goiás, has three chicken slaughter lines, and operates in two shifts, with a slaughter capacity of 176,000 birds, which have an average clean weight of 1.04 kg. Slaughterhouse E is located in the state of Paraná, has two chicken slaughter lines, operates in three shifts, and has a slaughter capacity of 475,200 chickens per day. The chicken slaughter line has a slaughter capacity of 17,600 birds per day with an average clean weight of 1.04 kg. From these carcasses, 144 samples of 25 g of skin and muscle were collected from the cloaca, neck, and wing regions. The samples of different regions of each carcass were grouped as established by Ordinance 1023/2024. Although this Ordinance does not specify whether samples should be collected from the ventral or dorsal side of carcasses, the samples were collected from the ventral side, as this procedure is commonly performed in slaughterhouses and their laboratories. This can be explained by the fact that the ventral surface of carcasses is naturally positioned toward the sampler when carcasses are hanging on the shackle line or placed on laboratory benches during sample preparation. This is also the reason why pooled ventral samples were selected for use in the second stage of our study, even though pooled dorsal samples showed higher *Enterobacteriaceae* counts than those collected from the ventral region.

In addition, 144 samples of neck skin (25 g, without muscle) were also collected from the carcasses. This region was chosen based on the results of Stage 1 of this study (see results). The *Enterobacteriaceae* counts of both groups of samples were performed as described below and then compared.

Although Ordinance 1023/2024 requires only the enumeration of *Enterobacteriaceae*, other indicator and pathogenic microorganisms were quantified in ten chicken carcasses using both excision methods. These counts were carried out solely for the purpose of verifying the microbiological trend and confirming whether both excision methods would yield the same results when considering other microorganisms, in addition to *Enterobacteriaceae.* The counts were performed in five poultry slaughterhouses located in different regions of Brazil, namely two in Paraná, one in Minas Gerais, one in Santa Catarina, and one in Mato Grosso. Sampling consisted of 25 g portions collected from the same carcasses tested by both excision methods. All samples were taken from the ventral portion of the carcasses and tested for the total mesophilic microorganisms, total coliforms, *E. coli*, and coagulase-positive *Staphylococcus*. The methods used are described below.

### 2.4. Microbiological Analyses

Each 25 g sample was mixed with 225 mL of 0.1% buffered peptone water (20 L bag, BBV, Bio BoaVista, Valinhos/São Paulo, Brazil) and homogenized for 1–2 min in a sample homogenizer (Model Smasher, AES Laboratoire, Biomérieux, Marcy-l’Étoile, France). Subsequently, each sample was serially diluted in 0.1% buffered peptone water, and aliquots were plated on different media according to the target microorganism. Enumeration of *Enterobacteriaceae* was performed following AFNOR 3M 01/16-09/97, while total mesophilic microorganisms, total coliforms, *E. coli*, and coagulase-positive *Staphylococcus* were determined according to AFNOR 3M 01/17-11/16, Microval 2017LR76, AFNOR 3M 01/09-04/03, respectively.

### 2.5. Statistical Analyses

Statistical analyses were performed using the R software 4.5.1 (2025-06-13 ucrt). After obtaining the mean, median, and standard deviation of the samples, the Shapiro–Wilk test was applied to check for the normality of data distributions and the Levene’s and f-test were applied to evaluate the homogeneity of variances, which are necessary assumptions for subsequent parametric methods. The Kruskal–Wallis test was used to compare more than two independent groups, and the Dunn’s test was applied as a post hoc test to identify differences between specific pairs of samples due to the absence of data normality. When samples showed normal distribution and homogeneous variances, Student’s *t*-test for independent samples was used to detect significant differences in the means between the groups. Finally, for comparisons between two independent samples without normal distribution, the non-parametric Wilcoxon rank-sum test was employed, which is appropriate for identifying differences in medians between groups with asymmetric distributions.

### 2.6. Economic Impact of the Different Excision Sampling Methods

The operational characteristics of 18 Brazilian poultry slaughterhouses were determined to assess the potential economic impact of excision sampling methods across diverse production realities. Specifically, the number of work shifts, chillers, and the mandated number of poultry carcasses for collection (in accordance with Ordinance 1023/2024) were identified and are presented in Table 1. This variation in process and production scale allows for a comprehensive evaluation of sampling economics in large-scale Brazilian poultry processing facilities. Our analysis focused on direct and quantifiable parameters to avoid indirect costs that are difficult to measure. Overhead, cold chain, recall probability, regulatory penalties, among others were not considered, as they are not consistently associated with excision sampling methods. A full economic assessment of all direct and indirect factors was beyond the scope of this part of the study. The economic impact of the two excision sampling methods was quantified considering the number of carcasses collected per day and per month to fully comply with Ordinance 1023/2024.

Net carcass weights were calculated based on established carcass meat yield and live weight parameters, resulting in a value of 1.04347 kg for light/grillers and 2.10854 kg for heavy broilers. Considering the total annual volume of broilers processed across the 18 slaughterhouses and the associated cumulative annual yield cost, the average cost of the harvested carcass (excluding giblets and feet) was estimated at approximately US $0.97 per kg. Subsequently, the economic impact (cost) of carcass sampling required to comply with Ordinance 1023/2024 [6] was determined. This calculation utilized the number of carcasses collected per day, the unit’s net weight production type (light/grillers and/or heavy broilers), and the derived value per kg, and was then compared with the cost associated with neck skin sampling. The calculations of these costs considered the mean cost of broiler produced by the slaughterhouses (US $0.97/kg). The mean carcass cost was derived from the company’s aggregated annual production volume and established carcass yield parameters for light (grillers) and heavy broilers. The total annual carcass yield cost was divided by the total annual kilograms produced, resulting in the average cost applied in the economic assessment.

Ordinance 1023/2024 mandates the number of carcasses to be collected hourly, per chiller, and per work shift in each Brazilian slaughterhouse. These collected carcasses must undergo *Enterobacteriaceae* counts. If the results meet the established satisfactory criteria (as discussed below), the required sampling frequency can be reduced. Therefore, Table 1 presents the calculated average number of carcasses collected per day under both the full sampling plan and the reduced sampling plan. Furthermore, Table 1 details the total number of carcasses to be collected across the 18 assessed slaughterhouses, determined by their respective number of lines, shifts, and chillers, assuming a standard 22 collection days per month.

### 2.7. Quantification of Time for Sample Preparation in Laboratory

The time required to perform sampling collection and preparation in laboratories was quantified for both excision methods The impact related to laboratory staff labor was quantified based on Man-Hours Worked (MHW). Specifically for the neck skin sampling method, the required time for collection of approximately 40 g of neck skin within the slaughterhouse processing area and the subsequent 25 g subsampling conducted in the laboratory was considered. To ensure a fair comparison, the common procedural steps shared by both methods—including the receipt of carcasses or samples in the laboratory, batch preparation, dilutions, and subsequent microbiological analyses—were excluded from the time quantification.

The calculation of total laboratory labor considered the time required for sample preparation to each method, as well as the time required for waste management. Waste management time included the duration to seal a filled garbage bag, replace it with a new clean plastic bag, and remove the waste from the laboratory to an appropriate disposal site. Waste disposal assumptions were established to standardize the calculation. For carcass sampling, it was assumed that one plastic bag was removed for every eight sampled chicken carcasses, based on laboratory practice of removing bags containing either 10 light/grillers or 5 heavy chicken carcasses. For neck skin disposal, the procedure was standardized to one trash bag per day, totaling 305 bags per year, accounting for the non-operational status of the laboratories on Sundays. Finally, sampling transportation time was not considered in the calculation, as the sample collection (carcasses or neck skins) and analysis laboratories were located within the same slaughterhouse facilities.

## 3. Results

The results of the *Enterobacteriaceae* counts for the skin and muscle portions from the wing, neck, and cloaca regions, as well as the pooled samples from the three regions of the bird carcasses positioned in the ventral and dorsal positions, are demonstrated in Table 2. To characterize the measurements obtained in the different regions evaluated, descriptive statistics were calculated, including measures of central tendency (mean and median) and dispersion (standard deviation).

The result of the Shapiro–Wilk test indicated that the wing (*p* = 0.0001) and cloaca (*p* = 0.026) samples did not exhibit a normal distribution (*p* < 0.05), necessitating the use of non-parametric statistical tests for subsequent analyses. In contrast, the neck (*p* = 0.180), ventral pooled (*p* = 0.090), and dorsal pooled (*p* = 0.720) samples showed normal distribution data (*p* > 0.05). Subsequently, Levene’s test was employed to evaluate the homogeneity of variances between the groups, based on this is a prerequisite for the validity of assumptions in parametric statistical methods. The test resulted in a *p*-value of 0.415 (*p* > 0.05), indicating that the variances of the groups were considered homogeneous. The Kruskal–Wallis test, a non-parametric alternative to analysis of variance (ANOVA), was used to compare independent groups due to the violation of the data normality assumption. The result showed a *p* = 0.052, a value slightly above the adopted level of significance (α = 0.05) for the comparison of the wing, neck, and cloaca samples with each other. Thus, no statistically significant difference was identified between the analyzed groups, indicating that the medians of *Enterobacteriaceae* counts of the groups did not differ.

Furthermore, Dunn’s multiple comparison test was employed to perform pairwise comparisons. It was observed that the comparisons between the wing and neck regions (*p* = 0.973), wing and cloaca (*p* = 0.116), and neck and cloaca (*p* = 1.000) confirmed the results of the previous test, indicating that the comparisons between the samples showed *p* > 0.05, confirming the absence of a significant difference in *Enterobacteriaceae* counts among the analyzed groups.

Additionally, the ventral and dorsal pooled samples were compared using Student’s *t*-test, as both groups exhibited a normal distribution of data. The result obtained was *p* = 0.0002 (*p* < 0.05), indicating a significant difference in the contamination between the ventral and dorsal regions.

According to Ordinance 1023/2024 [6], the regions of the chicken carcasses recommended for sample collection are the wing, neck, and cloaca. Considering that the standard carcass position during sampling, both in the industrial environment (carcasses hanging on the shackle line) and in the laboratory (carcasses on benches), is with the vent facing the collector, the standard sampling method was established as the skin and muscle pool obtained from the three mentioned regions, all located on the ventral face of the carcass.

To assess whether each of the recommended regions (wing, neck, and cloaca) could individually and representatively replace the commonly performed ventral pooled sampling, a comparative statistical analysis was performed between the ventral pooled collection and each of the other regions.

For this purpose, the Kruskal–Wallis test was used, which identified a *p* = 0.00001, indicating a statistically significant difference between the samples (*p* < 0.05). Considering a significance level of 0.05, it was observed that the comparisons between the dorsal and wing regions (*p* = 0.000002), dorsal and cloaca (*p* = 0.018), dorsal and neck (*p* = 0.0177416), and ventral and dorsal regions (*p* = 0.006) indicated statistically significant differences, demonstrating that the *Enterobacteriaceae* contamination on the dorsal region was higher than in the other evaluated parts. Conversely, all other comparisons had *p*-values greater than 0.05, such as the comparison between the ventral and wing regions (*p* = 0.842), ventral and neck (*p* = 1.000), ventral and cloaca (*p* = 1.000), wing and neck (*p* = 0.445), wing and cloaca (*p* = 0.434), and cloaca and neck (*p* = 1.000). Thus, the results indicate that, with the exception of the dorsal region pooled samples, which showed significantly higher contamination, the other regions do not exhibit significant differences in *Enterobacteriaceae* counts, with the observed variations attributable to sampling variability.

### 3.1. Comparison of Enterobacteriaceae Contamination According to the Two Excision Sampling Methods

The results of the *Enterobacteriaceae* counts for the pooled skin and muscle portions from the wing, neck, and cloaca regions, and the *Enterobacteriaceae* counts for neck skin are shown in Table 3.

The Wilcoxon rank-sum test revealed a statistically significant difference (*p* < 0.05) between the pooled samples and the neck skin samples (*p* = 0.00001), indicating that the latter showed higher *Enterobacteriaceae* contamination.

A comparative analysis of the *Enterobacteriaceae* counts from the neck skins of the 144 samples with the counts performed on 30 pooled samples collected from the dorsal region was carried out and is demonstrated in Table 4. The difference between sample sizes (n = 30 vs. n = 144) was considered in the statistical analysis. The nonparametric Wilcoxon test was used to compare the groups, because it is robust to unbalanced samples and its results are based on joint data ordering. However, it is recognized that the smaller sample size of the dorsal region implies reduced statistical power, which may limit the detection of subtle differences between distributions. Nevertheless, the inferential validity of the test remains preserved.

Wilcoxon rank-sum test revealed no statistically significant difference between the microbial counts of the pooled samples collected from the dorsal region and the neck skin samples (*p* = 0.453). Thus, it can be inferred that both exhibit similar *Enterobacteriaceae* load, suggesting comparable hygienic-sanitary conditions in these samples, since the counts were similar. Considering the similarity between the counts and the ease of carcass sampling within the slaughterhouse facilities, neck skin samples appear to be more suitable for evaluating the microbiological contamination of chicken carcasses.

Confirming the *Enterobacteriaceae* enumeration results, which demonstrated equivalence and microbiological representativeness between the two excision methods, the counts of total mesophiles, total coliforms, and *E. coli* obtained by both methods were also similar, showing no significant differences when analyzed by the *t*-test (*p* > 0.05). For example, the mean counts of total mesophiles using the ventral pooled method and the neck skin method were 3.28 ± 1.09 Log_10_ CFU g^−1^ and 3.73 ± 0.91 Log_10_ CFU g^−1^ (*p* = 0.311), respectively, while the mean counts of total coliforms were 1.8 ± 1.06 Log_10_ CFU g^−1^ and 2.09 ± 0.50 Log_10_ CFU g^−1^ (*p* = 0.431). The *E. coli* counts were 1.60 ± 0.94 Log_10_ CFU g^−1^ and 1.83 ± 0.50 Log_10_ CFU g^−1^ (*p* = 0.483). Coagulase-positive *Staphylococcus* counts were <1.0 Log_10_ CFU g^−1^.

### 3.2. Economic Impact

As can be observed in Table 1, the 18 large-scale Brazilian slaughterhouses analyzed operated in two or three shifts, had between one and three slaughter lines and chillers, and collected daily from 10 to 54 chicken carcasses to be analyzed according to Ordinance 1023/2024 [6]. The overall average total of carcasses collected in the 18 slaughterhouses was 573.82 per day or 12,624 carcasses per month.

Table 5 contains the estimated cost of discarded carcasses used for the pooled excision sampling (wing, neck, and cloaca regions) for compliance with Ordinance 1023/2024 [6], while Table 6 demonstrates the estimated cost using neck skin samples. As can be observed in these Tables, the monthly cost to analyze the 12,624.00 carcasses collected monthly was US $23,197.99 and the annual cost was US $278,375.89. In contrast, the monthly cost to analyze neck skins collected from the same number of carcasses was US $61.82, while the annual cost was US $727.08. These values represent a 99.73% reduction in the estimated cost if neck skins are sampled instead of whole carcasses, considering only the cost of the samples.

As can be seen in Table 7, the estimated labor time for cutting samples using the three-region grouped excision method and the neck skin excision method was 2600.54 and 925.76 h, respectively. The analysis steps were not considered because, regardless of the sampling method, they are performed in the same way. To determine the number of MHW, the average time was calculated for each activity: 1.03 min for cutting the parts and 0.37 min for cutting the neck skin. This average time was multiplied by the total number of samples taken in the year, which was 151,488.00. The removal of waste bags containing discards from both methods took 252.48 h for carcasses and 4.07 h for neck skin. To determine the time spent on waste removal, an average calculation was made of how many waste bags were removed to handle the disposal of carcasses and neck skin. An average of eight carcasses per waste bag was considered; therefore, the total number of carcasses handled in the year was divided by eight to determine the total number of waste bags (18,936.00 bags/year) and then multiplied by the average time spent closing a waste bag, discarding it, and replacing the disposal container with a new bag (0.8 min/waste bag). To calculate the amount of neck skin disposal, one garbage bag per day was considered and multiplied by the total number of working days in the year, which was considered to be 305 days. This value was then multiplied by the time for disposal and replacement of the garbage bags (0.8 min). The change in the collection method resulted in a total reduction in MHW by 67% per year, with 2853.02 h per year spent on cutting and disposing of carcasses and 929.83 h per year on neck skin.

## 4. Discussion

The investigation of *Salmonella*, *S. aureus*, mesophilic microorganisms, generic *E. coli*, and *Enterobacteriaceae* is commonly employed in poultry meat safety and quality programs. In Brazil, these microorganisms are monitored in chicken meat through various programs and regulations, which differ from Ordinance No. 1023/2024, requiring only *Enterobacteriaceae* counts in chicken carcasses to evaluate slaughterhouse performance and hygienic control of the slaughter process. For this reason, the present study assessed carcass excision sampling methods primarily using *Enterobacteriaceae* counts.

The results of the microbiological analyses demonstrated that the mean *Enterobacteriaceae* counts for the skin and muscle samples from the wing, neck, cloaca regions, and the ventral pooled samples were between 1.4 and 1.70 Log CFU/g and were all considered statistically similar. Other studies have presented similar *Enterobacteriaceae* counts in chicken carcasses or cuts. For example, De Villena et al. [4] showed that chicken carcasses and wings sampled post-chiller contained *Enterobacteriaceae* counts of 0.90 ± 0.08 Log CFU/mL and 1.64 ± 0.10 Log CFU/mL. Vargas et al. [5] found slightly higher *Enterobacteriaceae* counts in 71% of chicken carcasses also collected post-chilling, which were between 1.0 and 3.0 Log CFU/mL. The same authors demonstrated that 84% of the wing samples showed counts between 1.0 and 4.0 Log CFU/mL. Wyink et al. [3] investigated the contamination of skin from the back, neck, breast, abdominal region, and legs of chickens and concluded that there was no significant difference among the total viable microorganism counts.

According to Tortorello [12] and Saini et al. [13], the food industry has used counts of indicator microorganisms to evaluate the performance of hygienic-sanitary control when data on pathogenic microorganisms are scarce or difficult to collect in industrial environments. Brazil has acted in the same way in chicken meat production, and for this, the evaluation of the performance of carcass sampling methods is of great importance.

According to Ordinance 1023/2024 (Brazil, 2024), the result of the *Enterobacteriaceae* counts for each analyzed carcass sample must be expressed in Log CFU/g for the evaluation of the hygienic-sanitary performance of Brazilian slaughterhouses based on five-week rolling averages. Establishments considered satisfactory are those that present sample averages below 2.3 Log CFU/g, while establishments classified as having an acceptable performance are those that have up to 20% of the sample averages between 2.3 and 3.0 Log CFU/g (but no weekly average above 3.0 Log CFU/g). Finally, establishments considered to have an unsatisfactory hygienic-sanitary performance are those that present any weekly sample average above 3.0 Log CFU/g. If the mean counts obtained in the present study were maintained for five weeks and then submitted for this classification, the three sampled slaughterhouses would have been considered to have a satisfactory hygienic-sanitary performance.

The results of our study demonstrated that when skin and muscle were sampled separately from the wing, neck, and cloaca regions, and then compared with samples from the same three regions forming a pool of samples from the dorsal side of the carcasses, the dorsal sample pool showed significantly higher mean counts of *Enterobacteriaceae* than the separate parts. Similarly, when the contamination of neck skin-only samples (without muscle) was compared with the contamination from the pooled skin and muscle samples from the ventral region (n = 144), the neck skin showed significantly higher mean *Enterobacteriaceae* counts. The neck skin (without muscle) counts were statistically similar to the counts performed on the pooled skin and muscle samples from the dorsal region, which was considered more contaminated than the ventral region and from the individual parts. Within this context, the findings of this study demonstrate that neck skin excision is not inferior to the pooled ventral or dorsal excision method used to attend Ordinance 1023/2024. On the contrary, neck skin exhibited equal or higher *Enterobacteriaceae* counts than these pooled samples, indicating strong microbiological representativeness. These findings were corroborated by the counts of other indicator and pathogenic microorganisms tested in carcasses using both methods, which showed no statistically significant differences.

It is plausible that the skin of chicken carcasses is more contaminated than the muscle, since the skin is directly exposed to contamination sources such as equipment and utensil surfaces, other carcasses, residues, and even feces during slaughter, feather removal, evisceration, and cutting. The structure of chicken skin has fat, follicles, and folds that can retain dirt, feces, and microorganisms, making contamination removal difficult. The muscle, being under the skin, suffers less direct exposure to contamination sources and, therefore, tends to have a lower microbial load, especially if the chicken is handled correctly. In Brazil, large chicken slaughterhouses are automated and there is no manual handling by employees in the neck region. However, the neck skin may actually be more contaminated than other parts of the carcasses [3] due to its hanging position during the process, which allows the potable water used in washing to drain and carry microorganisms from other regions of the carcass to the neck. In addition, the back of the neck may also be more contaminated due to the automated evisceration process, which allows some viscera to touch the carcass when removed.

In the present study, the higher contamination of the neck skin samples and samples collected from the carcass back were significantly higher even than the contamination of the ventral pooled samples commonly collected in compliance with Ordinance 1023/2024 [6], which suggests that neck skin and dorsal region samples could better represent chicken carcass contamination when sampling carcasses by excision methods. These methods have been compared with other sampling methods, such as rinsing and swabbing of carcasses, and have shown similar performance to rinsing [7,8] or even better than rinsing and swabbing [11].

Considering the samples that can be collected to perform excision methods, neck skins appear to be among the most recommended because, in addition to showing counts similar to or higher than other carcass parts, as demonstrated in our study, they can be easily collected from carcasses hanging on shackle lines, inside the slaughterhouse facilities, which is not always possible when collecting multiple parts of ventral or dorsal samples. For example, an operator located in front of a hanging carcass can easily collect a neck skin sample, from both the ventral and dorsal regions, making only one excision, and this collection is faster and more representative of the carcass contamination than the pooled samples. Corroborating this data, Gill and Badoni [7] studied various chicken carcass sampling methods and reported that neck skin collection would be more convenient and less destructive than sampling by other methods, indicating it as the preferred method for enumerating bacteria on chicken carcasses.

### Cost Evaluation

Corroborating the preference for sampling chicken carcasses through neck skin excision, the cost evaluation of discarding carcasses used for pooled sampling versus neck skin also demonstrated an economic advantage for sampling the latter. For example, while the annual cost to analyze 12,624.00 carcasses, in 18 slaughterhouses in Brazil, using the pooled sample method, was approximately US $278,374.89 the cost to analyze the same number of neck skin samples was approximately US $727.08, which represents only 0.26% of the cost of the pooled samples. Neck skin collection can be performed directly on the process line, which is not the case with the collection of pooled samples that requires the carcass to be removed from the shackle line and transported to the laboratory or another suitable location to perform the other excisions. The cost of carcass transport is generally higher, as it needs more material for sampling and space to store sampled carcasses. Furthermore, the time required to collect from multiple carcass regions is significantly longer than the time to collect neck skin. As demonstrated in the results of the present study, the mean time to collect the three ventral regions of a carcass was 1.03 min, while the mean time to collect neck skin was 0.37 min. If the 151,488.00 carcasses collected in the 18 slaughterhouse facilities investigated in this study are considered, the mean time for the pooled sample collections would be 2600.54 h, while the mean time for the neck skin collections would be 925.76 h. The waste of animal protein was also much higher with carcass sampling for pooled sample collection. The same applies to the number of plastic bags and the labor involved in their removal. For example, while the removal of 18,936.00 bags containing sampled carcasses was estimated in one year across the 18 slaughterhouses, only 305 plastic bags were estimated for the removal of neck skin samples. The time of waste removal and labor for sample preparation was also expressively lower with neck skins, i.e., from 2853.02 to 929.83 h per year.

This information indicates significant advantages in adopting neck skin sampling, as it reduces sampling time, minimizes the waste of animal protein used in microbiological analyses by replacing cuts of higher commercial value, and lowers the costs associated with sampling and disposal materials. This easier sampling method may also enhance the effectiveness of corrective actions in the production process and improve the quality of the final product. It is important to emphasize that variations in indicator microorganism counts during process monitoring do not necessarily imply direct actions on the marketed final product, since samples are collected at intermediate stages of the process, prior to product completion and commercialization. It is well established that improvements in process quality reduce product blocks and recalls, whose financial impact varies with product type and market. For example, considering a scenario of a 10% increase in process monitoring effectiveness with a 5% reduction in blocks of a specific poultry product in a company producing 31,500,000 kg of this product monthly, with an average commercial value of US $2.16/kg and a block rate of 2% (630,000 kg), the estimated reduction would be 31,500 kg, representing an approximate monthly savings of US $68,727.27.

Based on these data, neck skin sampling for chicken carcass tests appears to be the most recommendable, as it provides equivalent or greater microbiological representativeness than other carcass regions while offering greater practicality and lower cost.

## 5. Conclusions

The results of the present study demonstrated that collection by excision of neck skin showed advantages compared to collection by excision from different parts of the chicken carcass. When neck skin was collected together with muscle and then compared with skin and muscle from other regions, the *Enterobacteriaceae* counts were statistically similar. However, when counts from neck skin alone were compared with counts from skin and muscle of three ventral carcass regions, as established by Regulatory Ordinance 1023/2024 (Brazil, 2024), neck skin counts were significantly higher. Neck skin samples showed counts similar to samples collected from the dorsal side of the carcasses, a region that presented higher contamination than the ventral region of the carcasses. The results of this study demonstrate that neck skin excision exhibited equal or higher *Enterobacteriaceae* counts than the pooled samples, indicating microbiological representativeness. Further, neck skin collection was faster, easier to perform, generated less waste, and was expressively cheaper than the pooled sample collection. These results indicate that collection by neck skin excision offers several advantages when compared to collection by excision from multiple regions of chicken carcasses, whether ventral or dorsal.

## Figures and Tables

**Table 1:** Description of 18 Brazilian poultry processing plants, their number of shifts, chiller, sample averages, total carcasses and weight (kg), reduced or full sampling plan according to Ordinance 1023/2024.

Plant	Shifts (n)	Chiller Lines	Carcass/Day (Full Sampling) ^2^	Carcass /Month (Full Sampling)	Carcass/Day (Reduced Sampling) ^3^	Carcass /Month (Reduced Sampling)	Clean Weight /Month (Full Sampling) (kg)	Clean Weight /Month (Reduced Sampling) (kg)
1 ^1^	2	1	16	352	4.36	96	367.30	100.17
2 ^1^	2	3	48	1056	13.09	288	1101.90	300.52
3	2	2	32	704	8.73	192	1484.41	404.84
4	2	2	32	704	8.73	192	1484.41	404.84
5	2	1	16	352	4.36	96	742.21	202.42
6	2	3	48	1056	13.09	288	2226.62	607.26
7	2	1	4.36	96	4.36	96	742.21	202.42
8 ^1^	3	3	72	1584	20.36	448	1652.85	467.47
9	3	2	48	1056	13.09	288	2226.62	607.26
10	2	2	32	704	8.73	192	1484.41	404.84
11 ^1^	2	3	48	1056	13.09	288	1101.90	300.52
12	2	2	32	704	8.73	192	1484.41	404.84
13	2	2	32	704	8.73	192	1484.41	404.84
14	2	1	16	352	4.36	96	742.21	202.42
15	2	2	8.72	192	8.73	192	1484.41	404.84
16	2	3	48	1056	13.09	288	2226.62	607.26
17	2	2	32	704	8.73	192	1484.41	404.84
18 ^1^	3	1	8.72	192	8.73	192	550.95	200.35
Total			573.8	12,624	173.09	3808	24,072.28	6631.95

^1^ Griller processors. ^2^ Average number of carcasses collected per day according to the full sampling plan. ^3^ Average number of carcasses collected per day according to reduced sampling plan.

**Table 2:** Descriptive statistics of *Enterobacteriaceae* counts from wing, neck, and cloaca samples collected from chicken carcasses and from pooled samples of these regions collected from the ventral and dorsal sides of the carcasses.

Sample	Mean Log_10_ CFU g^−1^	Median Log_10_ CFU g^−1^	Standard Deviation
Wing	1.43	1.30	0.491
Neck	1.69	1.70	0.451
Cloaca	1.71	1.70	0.580
Ventral	1.67	1.65	0.481
Dorsal	2.24	2.22	0.613

**Table 3:** Descriptive statistics of *Enterobacteriaceae* counts from pooled samples collected from the ventral region of chicken carcasses and from neck skin samples collected from the ventral and dorsal regions of the carcasses.

Sample	Mean Log_10_ CFU g^−1^	Median Log_10_ CFU g^−1^	Standard Deviation
Ventral pool	1.93	1.87	0.733
Neck skin	2.37	2.30	0.837

**Table 4:** Descriptive statistics of *Enterobacteriaceae* counts from pooled samples collected from the dorsal region of chicken carcasses and from neck skin samples collected from the ventral and dorsal regions of the carcasses.

Sample	Mean Log_10_ CFU g^−1^	Median Log_10_ CFU g^−1^	Standard Deviation
Dorsal pool (n = 30)	2.24	2.22	0.613
Neck skin (n = 144)	2.37	2.30	0.837

**Table 5:** Estimated cost of carcasses for pooled excision sampling to comply with Ordinance 1023 in 18 Brazilian slaughterhouses.

Number of Carcasses per Month	Weight (kg) per Month	Estimated Cost per kg	Estimated Cost per Month	Estimated Cost per Year
12,624	22,102.32	US $1.04	US $23,197.99	US $278,375.89

**Table 6:** Estimated cost for sampling by excision of chicken neck skin in 18 Brazilian large-scale slaughterhouses.

Number of Carcasses per Month	Weight (kg) per Month of Neck Skin	Estimated Cost per kg	Estimated Cost per Month	Estimated Cost per Year
12,624	504.96	(US $0.12) ^1^	(US $60.59) ^1^	(US $727.08) ^1^

^1^ The estimated cost per kilogram of neck skin (US $0.12/kg) was calculated based on the average carcass value (US $0.97/kg), in proportion to the average mass of 40 g of neck skin collected per carcass.

**Table 7:** Estimated labor times based on man-hours worked in the laboratory for sample preparation by the two excision methods in 18 Brazilian slaughterhouses.

Procedure	Time (min.)	Carcasses Sampled per Year	Bags Removed per Year	Worked Hours per Year
Excision sampling from three carcass regions (pool)	1.03	151,488.00		2600.54
Neck skin excision	0.37	151,488.00	-	925.76
Removal of waste bags containing carcasses	0.80	-	18,936.00 ^1^	252.48
Removal of waste bags containing neck skins	0.80	-	305 ^2^	4.07

^1^ Each bag with eight carcasses. ^2^ Day worked in the laboratory where a bag of neck skin was removed.

## Data Availability

The original contributions presented in this study are included in the article. Further inquiries can be directed to the corresponding author.
